# Chemical reprogramming regulates Tip60 expression to improve cleavage rates in somatic cell nuclear transfer reconstituted embryos of cashmere goats

**DOI:** 10.3389/fvets.2025.1720533

**Published:** 2025-12-19

**Authors:** Xiaoshu Zhe, Lihui Zhang, Rui Ding, Hairui Ma, Yaoguang Zhang, Fei Hao, Dongjun Liu, Yang Li

**Affiliations:** 1 State Key Laboratory of Reproductive Regulation and Breeding of Grassland Livestock, School of Life Sciences, Inner Mongolia University, Hohhot, China; 2 Department of Cell Biology and Stem Cell Research Center, School of Basic Medical Sciences, Peking University, Beijing, China

**Keywords:** cashmere goat, chemical reprogramming, histone acetylation, small molecule compounds, somatic cell nuclear transfer

## Abstract

The low efficiency of somatic cell nuclear transfer (SCNT) severely limits its application in animal cloning and regenerative medicine. To address this core scientific challenge, this study aims to explore a chemical reprogramming strategy that enhances the division rate of SCNT embryos during early developmental stages prior to transfer by pre-treating donor cells. Leveraging the role of small molecules in regulating cellular reprogramming, we designed a combination of small-molecule compounds (including 8 μM TranylcyprominT, 5 μM EPZ00477, 400 μM VPA, 8 μM Repsox, 1.2 μM PD0325901, 0.4 μM CHIR99021, 0.2 μM DZNeP, 8 μM Y-27632, and 1.2 μM UNC) to pre-treat donor cells, followed by embryo reconstruction and *in vitro* culture. Results demonstrated that this chemical treatment significantly improved embryo cleavage rates (35.59% vs. 46.15%). The combination of small molecules significantly upregulates the expression of core pluripotency genes (NANOG, SOX2, OCT4) and histone acetyltransferase TIP60 in donor cells. In summary, this study not only demonstrates the efficacy of chemical reprogramming in enhancing the early developmental capacity of SCNT embryos in large mammals but also lays a solid foundation for further elucidating its molecular mechanisms.

## 1 Introduction

Somatic cell nuclear transfer (SCNT) technology presents significant application potential in the field of genetic breeding for large mammals. However, its cloning efficiency remains generally low, with average success rates typically ranging between 0.3 and 2% (1). Despite numerous efforts to improve its efficiency in recent years, SCNT efficiency in large mammals such as pigs (2) and sheep (3) has only improved to 1–5%. In addition, the successful birth of cloned animals is still accompanied by numerous abnormal phenotypes, including macrosomia, muscular deformities, organ developmental abnormalities, and placental dysfunction (4). These issues severely limit the further application and development of this technology.

In recent years, extensive reports have focused on the use of small molecules to induce cellular reprogramming in human (5) and mouse (6) cells. Theoretically, chemical reprogramming systems could be applied to large livestock; however, research on the use of chemically induced reprogramming to enhance cloning efficiency in large animals remains relatively scarce. Small molecules offer multiple advantages, including good cellular permeability, non-immunogenicity, ease of storage and standardization, and low cost (7). Moreover, these molecules can directly target intracellular and cell surface proteins involved in cellular signaling and epigenetic regulation, thus providing unique advantages over genetic manipulation approaches (8). In 2013, Science published a landmark study by the team of Hongkui Deng at the Stem Cell Research Center of Peking University. These authors used a combination of small molecules (VPA, CHIR99021, 616452, tranylcypromine, forskolin, DZNep, and TTNPB) to reprogram mouse somatic cells into induced pluripotent stem cells (iPSCs), which are termed chemical-induced pluripotent stem cells (CiPS) (9).

In addition, intervening in the epigenetic state of donor cells via small molecules to enhance nuclear transfer efficiency has become a common research strategy in recent years. For instance, treating donor cells with trichostatin A or valproic acid increases low histone acetylation levels (10); similarly, using G9a inhibitors to reduce reprogrammed histone methylation levels enhances the developmental capacity of cloned embryos (11). The chemical small molecules CHIR99021, 616,452, TTNPB, JNKIN8, and 5-azacytidine synergistically regulate intracellular signaling pathways and induce epigenetic modifications to reprogram human adult cells into induced pluripotent stem cells (12). The chemical small molecules E616452, AM580, and CHIR99021 synergistically induce efficient conversion of mouse embryonic stem cells into embryonic foundation cells (13). The small molecule compound scriptaid enhances goat embryo reprogramming efficiency by modulating the expression of pluripotency-related molecules in donor cells (8). Although research on this topic is becoming more prominent in animal studies, it primarily remains in the preliminary exploration phase.

This study used an established library of small molecules for epigenetic regulation and integrated core reprogramming molecular combinations and reagent sets reported in chemical reprogramming systems (12, 14, 15). Nine small-molecule compounds with the potential to promote reprogramming were selected as research subjects. We then artificially regulated the epigenetic state and *in vitro* growth characteristics of goat fetal fibroblast cells (gFFCs). The effects of the small-molecule compound combinations on reconstituted embryos were investigated by observing changes in cellular reprogramming capacity and their impact on SCNT-reconstructed embryo development. The aim of our research was to provide a theoretical foundation for elucidating the objective mechanisms underlying the influence of small molecules on SCNT-reconstructed embryo development and enhancing the developmental efficiency of cloned embryos.

## 2 Methods

### 2.1 Cell lines and cultures

The cell lines used in this study were goat fetal fibroblast cells (gFFCs), which were isolated and preserved in a liquid nitrogen tank in the laboratory and used as nuclear donor cells for SCNT. gFFCs were cultured in a DMEM/F12 culture medium containing 10% fetal bovine serum at 37 °C and 5% CO_2_, and the culture medium was changed once every 2 d. When adherent cell confluence reached 80–90%, the cells were washed twice with DPBS, digested with 0.25% trypsin for 3 min, and passaged. The culture reagents were purchased from Vivacell (Shanghai, China). All experiments were conducted following the National Research Council Guidelines for the Care and Use of Laboratory Animals. The animal research protocol was approved by the Animal Care and Use Committee of Inner Mongolia University (protocol code: IMU-GOAT-2022-021). The animal samples were obtained from the Inner Mongolia Yi-Wei White Sheep and Cashmere Goat Limited Liability Company.

### 2.2 Preparation and use of small molecule compounds

The working concentrations for all small molecules in this study were primarily determined with reference to the concentration ranges recommended for cell culture experiments by the respective manufacturers’ reagent manuals. Gradient concentrations were tested in preliminary experiments to establish the final doses used. All small molecule compounds were first prepared as stock solutions and then diluted to the desired working concentrations using our basal culture medium (specifically, DMEM/F12 culture medium containing 10% fetal bovine serum) before application to the cells. Following the compound treatment period, the medium containing the compounds was removed. The cells were then washed and subsequently cultured in fresh complete DMEM/F12 medium supplemented with 10% fetal bovine serum until further analysis or endpoint measurement.

### 2.3 CCK-8 toxicity assay

Drug treatment were performed when the cell confluency reaches 60%. The concentration gradient for small molecule compounds should be designed according to the reagent manual. Please refer to the Supplementary Table S2 for the catalog numbers of the compounds. The different concentration gradients were applied for 48 h. Cells treated with a volume and concentration of DMSO equal to that used in the small molecule-treated groups were designated as the 0 μM control. Cells were inoculated in 96-well plates, and when the cells were completely attached to the wall and reached 60% confluency, the concentrations and compounds of the culture medium were changed. Subsequently, 10 μL of CCK-8 reagent (Yeasen, Shanghai, China) was added to each well at the specified treatment time, and the cells were incubated for 2 h. The optical density was determined at 450 nm using enzyme labeling (Thermo Fisher Scientific, Waltham, MA, United States).

### 2.4 RNA extraction and qRT-PCR

Total RNA was extracted from the cells using RNAiso (Yeasen, Shanghai, China), and cDNA was synthesized using a gDNA Removal Kit (Takara) following the manufacturer’s instructions. cDNA was amplified for reverse transcription using TB Green^®^ Premix Ex Taq^™^ II (Takara) and assessed using LightCycler 96 (Roche) for gene expression. Relative gene expression levels were calculated using the comparative CT method (2^−ΔΔCT^) with glyceraldehyde 3-phosphate dehydrogenase (GAPDH) as the housekeeping gene. Primers used in this study are listed in Supplementary Table S1.

### 2.5 Protein extraction and western blotting

Cell samples were extracted using the Mammalian Protein Extraction Kit (CWBIO), and the protein concentration was determined using the BCA Protein Assay Kit (Thermo Fisher Scientific). Protein expression was detected through immunoblotting using *α*-tubulin as an internal reference. Proteins were separated using sodium dodecyl-sulfate polyacrylamide gel electrophoresis and electrophoretically transferred onto nitrocellulose membranes (Pall), which were closed with 5% skimmed milk at room temperature and incubated with primary antibody (1:1,000) overnight at 4 °C. After washing three times with TBST, the membranes were incubated with a horseradish peroxidase-coupled secondary antibody (1:10,000) for 1 h at room temperature. Antigen–antibody complexes were visualized using Pierce ECL protein blotting substrate (Thermo Fisher Scientific), and membrane images were acquired using a Tanon 4800 microscope and analyzed in grayscale using ImageJ software.

### 2.6 Somatic cell nuclear transfer

Cashmere goat ovaries were collected from a slaughterhouse, and the follicles on the ovary surface were manually scratched off to collect the cumulus-oocyte complexes (COCs). The COCs were transferred to a preheated oocyte maturation medium *in vitro* using an oral pipette and cultured in a cell culture incubator with 5% CO_2_ at 38.5 °C for 20–22 h. The mature oocyte was enucleated via micromanipulation to produce a cytoplast. Subsequently, a donor cell was microinjected into the perivitelline space of the cytoplast. After *in vitro* maturation, oocytes were denuded and the 1st polar body and oocyte nuclei were removed using micromanipulation. Oocytes and donor cells (gFFCs) were microinjected into the vitelline membrane gap of zona-less oocytes and then incubated in maturation culture droplets for 20–30 min. Subsequently, electrical fusion was performed using an ECM 2001 Electro-Cell Manipulator (Harvard Apparatus). The reconstructed embryos were placed in a fusion solution consisting of 0.28 M mannitol, supplemented with 0.1 mM MgSO₄ and 0.05 mM CaCl₂. A single electrical pulse of 90 V was applied for 30 μs in the electrode chamber. Reconstructed embryos were pretreated in synthetic oviductal fluid containing 5 M A23187 amino acids (SOFaa solution) for 5 min and transferred to SOFaa solution containing 2 mM 6-dimethylaminopurine for 3.5 h. Reconstructed embryos were cultured for *in vitro* development in a cell culture incubator at 38.5 °C and 5% CO_2_. After 48 h of culture, the cleavage rate of the embryos was determined, and the embryo morphology was observed under a stereomicroscope. The reagents were purchased from Sigma-Aldrich.

### 2.7 Statistical analysis

The experimental data were statistically analyzed using GraphPad Prism 10 and expressed as the mean ± standard deviation. Differences between two groups were assessed using an unpaired two-tailed Student’s *t*-test, whereas differences between multiple groups were compared based on one-way ANOVA with Tukey’s *post hoc* test. Significance differences were determined at follows: ns > 0.05, ^*^p < 0.05, ^**^p < 0.01, ^***^p < 0.001, and ^****^p < 0.0001. All experiments were independently repeated three times.

## 3 Results

### 3.1 Screening of chemical small molecule compounds for optimal treatment concentration in gFFCs

**Figure 1:**
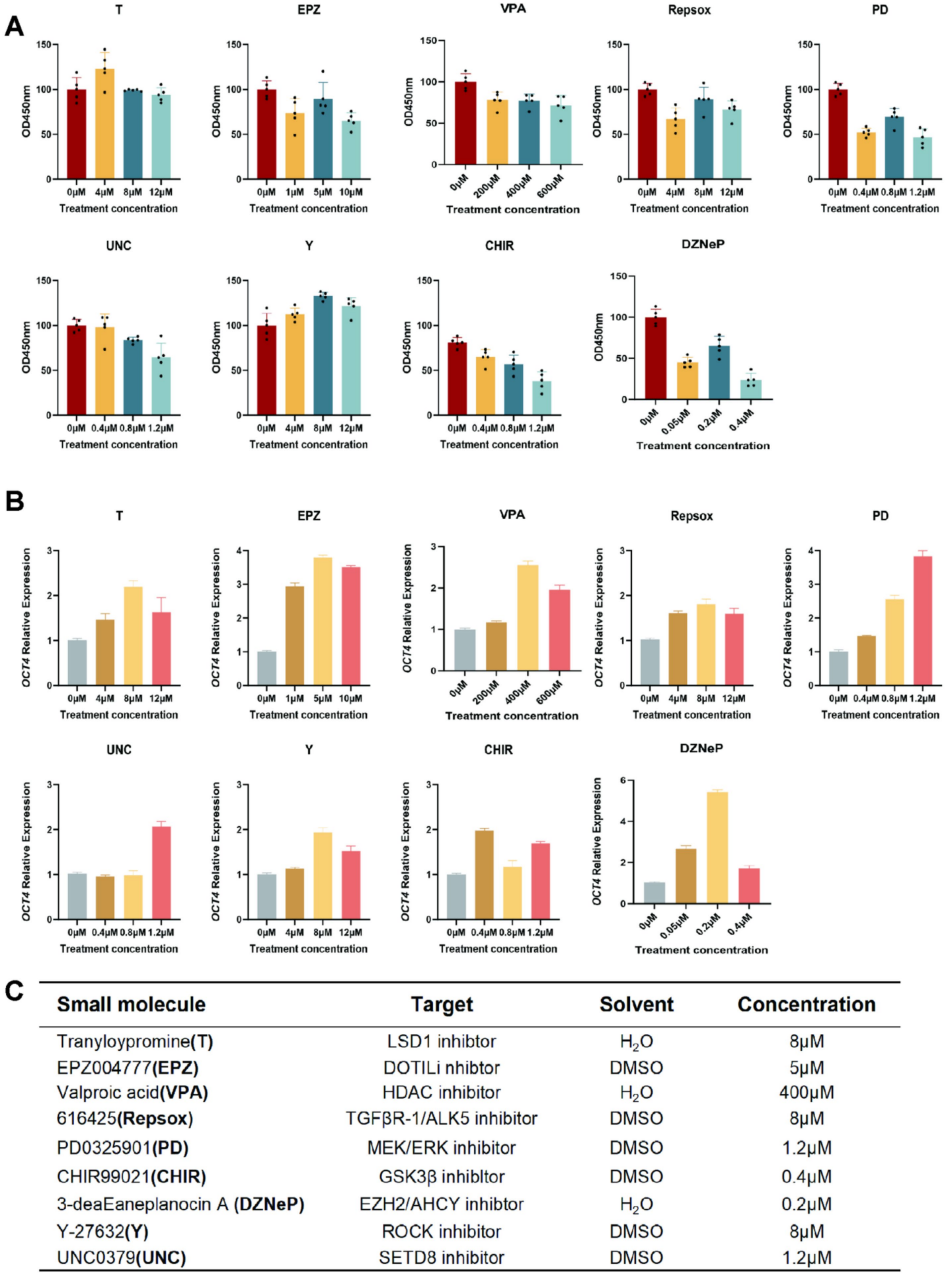
Screening of small molecule compounds for optimal treatment concentration and time. **(A)** A cell counting kit 8 assay was used to detect the effect of small molecule compounds on the growth activity of gFFCs, the *x*-axis represents the compound treatment concentration, and the *y*-axis represents the cell survival rate. **(B)** Detection of *OCT4* expression using quantitative real-time polymerase chain after treatment with the compounds used in the final experiment. **(C)** Concentrations of small molecule compounds used in the final experiment. Data (*n* ≥ 3) are represented as the mean ± SD.

**Figure 2:**
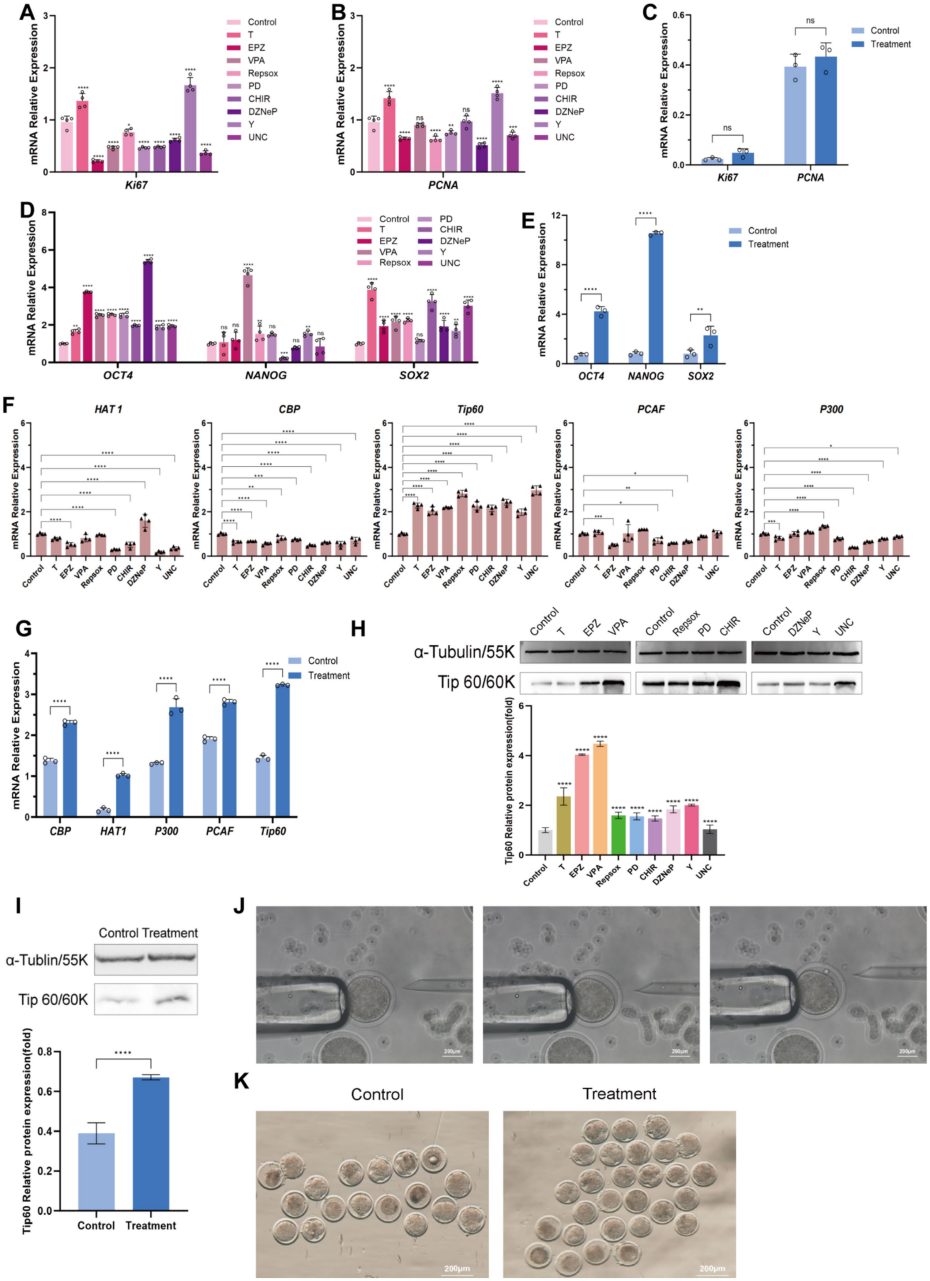
Effects of the small molecule compounds on gFFCs. **(A–C)** qRT-PCR was used to detect the expression of Ki67 and PCNA after treatment of gFFCs with small molecule compounds and their combinations. **(D,E)** qRT-PCR analysis showing the expression of pluripotency factors after treatment of gFFCs with small molecule compounds and their combinations. **(F,G)** qRT-PCR analysis showing the expression of HAT-related genes after treatment of gFFCs with small molecule compounds and their combinations. **(H,I)** WB analysis of the expression of Tip60 proteins after treatment of gFFCs with small molecule compounds and their combination grayscale analysis. **(J)** SCNT process. **(K)** SCNT-reconstituted embryo cleavage morphology. Data (*n* ≥ 3) are represented as the mean ± SD; ns > 0.05, ^*^p < 0.05, ^**^p < 0.01, ^***^p < 0.001, and ^****^p < 0.0001.

In this study, gFFCs were used as a model to determine the optimal treatment concentration of nine small molecule compounds, namely, tranylcypromine (T), EPZ004777 (EPZ), valproic acid (VPA), 616,452 (Repsox), PD0325901 (PD), CHIR99021 (CHIR), 3-deazaneplanocin A (DZNeP), Y-27632 (Y), and UNC0379 (UNC). Four concentration gradients with a treatment time of 48 h were set for each compound, with 0 μM as the control. First, the effects of different small molecule compounds on cell activity after cell treatment were detected using the cell counting kit 8 assay (Figure 1A). T and Y had a growth-promoting effect on cell growth. EPZ, VPA, Repsox, PD, CHIR, DZNeP, and UNC exhibited varying levels of cytotoxicity toward the cells and inhibited cell activity. *OCT4* is a crucial maternal primary effector gene in early embryo development. In this study, mRNA expression level changes of *OCT4* were used as a criterion for screening the concentration of small molecule compounds. All nine small molecule compounds promoted *OCT4* expression to different degrees (Figure 1B). Based on these results, we screened the concentrations of nine molecular compounds that had a relatively small effect on cell activity and a significant effect on *OCT4* expression as the optimal conditions for treating cells in subsequent studies (Figure 1C). The specific concentrations were as follows: 8 μM T, 5 μM EPZ, 400 μM VPA, 8 μM Repsox, 1.2 μM PD, 0.4 μM CHIR, 0.2 μM DZNeP, 8 μM Y, and 1.2 μM UNC. Based on the aforementioned treatment regimen, we employed real-time quantitative PCR technology to assess the effects of small-molecule compounds and their combinations on the transcriptional levels of the two proliferation markers *Ki67* and *PCNA* in gFFCs. Cells treated with a volume and concentration of DMSO equal to that used in the small molecule-treated groups were designated the control group. The results showed that after treatment with nine individual small-molecule compounds (Figures 2A,B), T and Y significantly upregulated *Ki6*7 and *PCNA* expression, suggesting their potential involvement in promoting cell proliferation. However, other small-molecule compounds significantly downregulated *Ki67* expression. Moreover, VPA and CHIR had no significant effect on *PCNA* expression, whereas EPZ, Repsox, DZNeP, and UNC significantly downregulated *PCNA* expression, indicating that these small-molecule compounds may have an inhibitory effect on cell proliferation. Compared with the DMSO control group, the expression levels of *Ki67* and *PCNA* did not show significant changes after treatment with the combination of small-molecule compounds (Figure 2C), indicating that this combination had no obvious short-term effect on cell proliferation.

### 3.2 Chemical small molecule compounds modulate core pluripotency marker expression to promote cellular reprogramming

*OCT4*, *NANOG*, and *SOX2* are the core markers of pluripotency, which are crucial factors in cell reprogramming and play crucial roles in maintaining pluripotency (16). We performed qRT-PCR to examine the effects of combinations of small molecule compounds on the transcript levels of *OCT4*, *NANOG,* and *SOX2* in gFFCs. The results showed that among the nine small-molecule compounds used to treat gFFCs, DZNeP induced the most significant upregulation of *OCT4* expression (Figure 2D). In addition, VPA and Repsox both significantly upregulated *NANOG* expression, while Y showed a similar trend. Notably, CHIR significantly downregulated *NANOG* expression in gFFCs. Eight small-molecule compounds significantly upregulated *SOX2* expression; however, PD showed no obvious effect. Further experiments indicated that the combination of small-molecule compounds significantly upregulated *OCT4, NANOG,* and *SOX2* expression, with particularly significant changes in *NANOG* compared to the DMSO group (Figure 2E).

### 3.3 Chemical small molecule compounds affect histone acetyltransferase Tip60 expression in gFFCs

Histone acetylation is a crucial epigenetic modification during early embryonic development and reprogramming (17), and the acetylation capacity of nuclear donor cells is essential for the late development of reconstructed embryos (18). We performed qRT-PCR to examine changes in the expression of histone acetyltransferase (HAT)-related genes (*HAT1*, *CBP*, *Tip60*, *PCAF*, and *P300*) in gFFCs treated with a combination of small molecule compounds. Transcriptional level differences (Figure 2F) showed that *Tip60* expression was significantly upregulated after treating gFFCs with nine different small-molecule compounds. Compared with the DMSO control group, the combined use of small-molecule compounds significantly upregulated Tip60 expression (Figure 2G). Western blot analysis revealed that Tip60 expression levels in the treatment groups were significantly higher than those in the DMSO control group (Figures 2H,I), which is consistent with the transcriptional level data. These findings suggest that these small molecules may regulate Tip60 protein expression, thereby altering histone acetylation levels in nuclear donor cells.

### 3.4 Treatment of donor cells with combinations of chemical small molecule compounds affects the rate of cleavage in reconstituted embryos

To investigate the effects of small molecules on the developmental reprogramming of SCNT-derived embryos, we treated donor oocytes with combinations of small molecules prior to SCNT-based embryo reconstruction. This was followed by somatic cell nuclear transfer procedures, including oocyte enucleation, donor cell preparation and aspiration, and nuclear transfer (Figure 2J). Forty-eight hours after SCNT, we observed and recorded the cleavage stage number and developmental progress of the embryos. In this study, ≤350 oocytes were collected per experiment, with an *in vitro* maturation rate of ≤88 oocytes (approximately 25%). Regarding the SCNT embryo reconstruction outcomes (Table 1), the untreated and chemically treated groups yielded 165 and 169 successfully fused embryos, respectively, among which 59 and 78 embryos underwent cleavage, respectively. Statistical analysis revealed that treatment with small molecules increased the cleavage rate of reconstructed embryos by 10.56%. Specifically, 12 embryos in the untreated group reached the 2-cell stage and 9 reached the 4-cell stage, although none progressed to the 8-cell stage. In contrast, 18 embryos in the chemically treated group reached the 2-cell stage and 14 reached the 4-cell stage. Notably, four embryos in the compound-treated group advanced prematurely to the 8-cell stage (Figure 2K), a phenomenon not observed in the blank control group. Statistical analysis showed that compared to the control group, the compound-treated group exhibited an increase in the cleavage rate from 20.34 to 23.08% at the 2-cell stage, from 15.25 to 17.95% at the 4-cell stage, and from 0 to 5.13% at the 8-cell stage. This suggests that small-molecule compounds may slightly increase early embryonic development.

**Table 1:** Statistics on the cleavage rate of SCNT embryos.

Treatment	Constructed embryos (No.)	Cleaved embryos (%)	2-cell rate (%)	4-cell rate (%)	8-cell rate (%)
Control	165 (±11)	59 (±5) 35.59% (±2.15%)^a^	12 (±5) 20.34% (±1.98%)	9 (±5) 15.25% (±0.64%)	0 (±0) 0% (±0%)
Treatment	169 (±9)	78 (±7) 46.15% (±1.35%)^b^	18 (±5) 23.08% (±2.05%)	14 (±5) 17.95% (±1.19%)	4 (±5) 5.13% (±1.01%)

Cleavage rate, cleaved embryos/reconstructed embryos; 2-cell rate, 2-cell/cleaved embryos; 4-cell rate, 4-cell/cleaved embryos; 8-cell rate, 8-cell/cleaved embryos. Within the same column, different superscripts indicate significant differences at *p* < 0.05.

## 4 Discussion

To date, various animals have been born using SCNT technology, such as cloned cattle and mice. These animals show unique value and broad application prospects in the preparation of animal models and modern biotechnological breeding of livestock. However, the developmental potential of SCNT embryos remains low. Several limitations need to be addressed in the process of reprogramming differentiated somatic cells to the totipotent state of oocytes to markedly improve SCNT efficiency.

**Figure 3:**
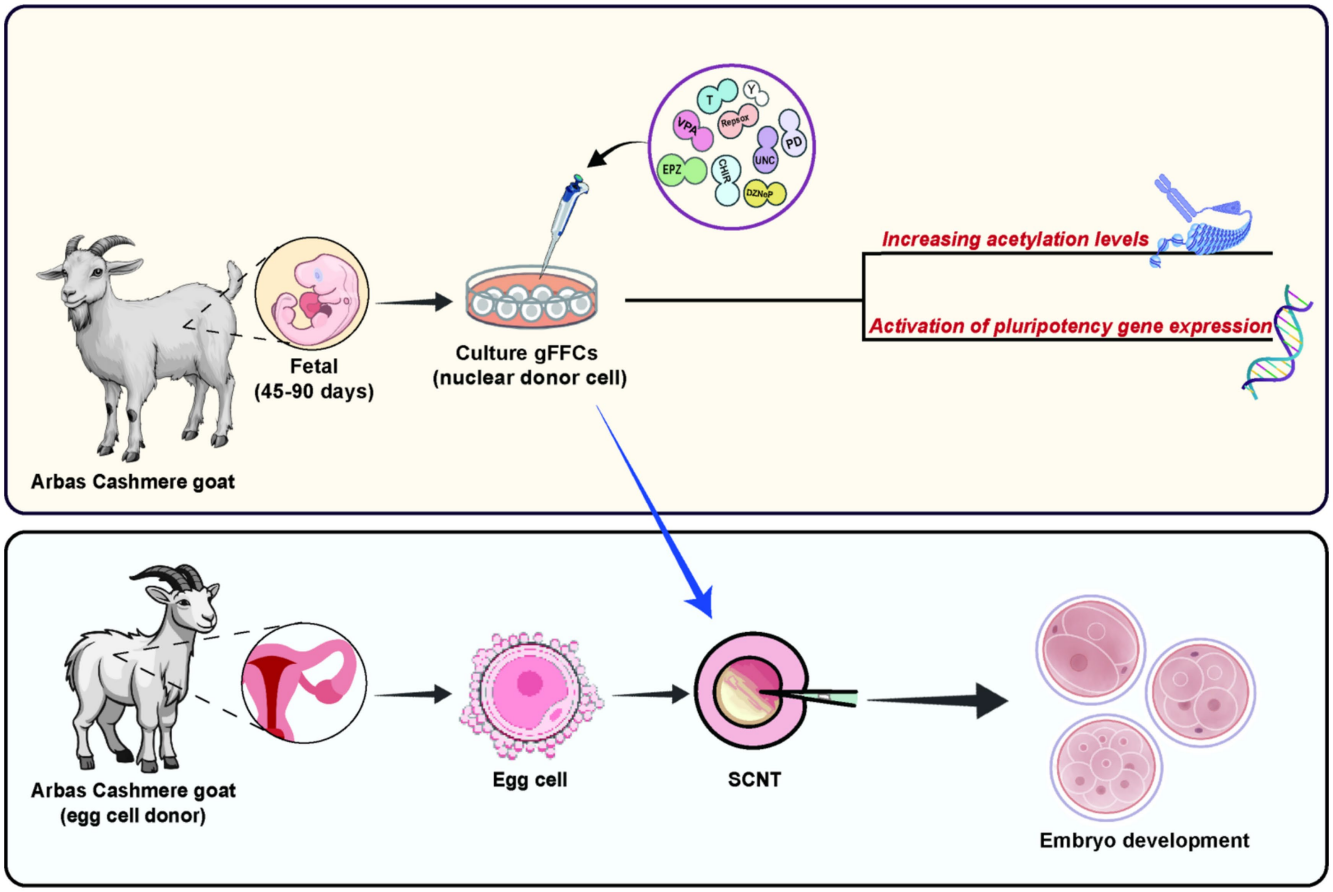
Summary of this study.

The results of this study confirm that chemical small-molecule reprogramming treatment can significantly regulate the reprogramming process of donor cells from cashmere goats and activate the expression of core pluripotency gene networks (*NANOG, SOX2, OCT4*). These findings provide compelling preliminary evidence for enhancing the reversal of somatic cells to a pluripotent state. The small molecule compounds used in this study increased histone acetylation levels in donor cells, significantly enhanced the expression of pluripotency core genes, and effectively increased the division rate of reconstructed embryos (Figure 3). This study provides a new experimental basis for investigating how small molecule compounds affect reprogramming and reveals new research directions for improving the efficiency of SCNT.

In this study, we used several epigenetic modulators and signaling pathway inhibitors, including histone demethylase (LSD1) inhibitor T, HDAC inhibitor VPA, histone methyltransferases of DOT1L, EZH2, SETD8 inhibitor EPZ, DZNeP, UNC, TGF-*β* receptor inhibitor Repsox, MEK/ERK signaling pathway inhibitor PD, GSK-3β inhibitor CHIR, and ROCK signaling pathway inhibitor Y (12). Compared to the distinct effects of individual compounds on cells, small-molecule combinations demonstrate greater efficacy. This experimental finding aligns with previous research (19) and indicates that drug combinations can enhance reprogramming outcomes. Chemical small molecule compounds can directly target intracellular and cell-surface proteins to regulate signaling and epigenetic modifications. Combinations of exogenously synthesized small molecule compounds can remodel cellular gene regulatory networks through multi-target synergistic effects (20). Despite the possible toxicity and other side effects of chemical small molecule compounds, their structural and functional diversity is almost unlimited. Moreover, their potential for application through rational design and synthetic chemistry optimization cannot be underestimated (21).

The acetylation capacity of nuclear donor cells is essential for the late development of reconstructed embryos (18). Histone hyperacetylation upregulates reprogramming transcription factors by relaxing chromatin and enhancing its accessibility (22). In the present study, treatment with small molecule compounds significantly upregulated histone acetylase Tip60, which increased histone acetylation and promoted chromatin opening and gene transcriptional activation.

*NANOG*, *SOX2*, and *OCT4* form a positive feedback regulatory network (23) that regulates each member’s expression and represses differentiation-related gene expression, which reverts differentiated cells to a pluripotent state. Chemical reprogramming promotes pluripotency and *in vitro* totipotency at different early developmental stages and affects cell differentiation potential and embryonic development (24). In the present study, a combination of small molecule compounds prematurely activated *OCT4*, *NANOG*, and *SOX2* expression in donor cells. This factor could play a pivotal role in increasing the rate of egg cleavage in reconstituted embryos.

In our study, treatment with small-molecule compounds significantly improved the overall cleavage rate of cloned embryos (from 35.59 to 46.15%). Quantitatively, the number of cleaved embryos increased from 59 to 78 with chemical treatment, with some even developing to the 8-cell stage. This 10.56% improvement represents a highly significant advancement in large mammalian embryo research. Moreover, it indicates that the primary function of small molecules is not to accelerate transition rates at specific stages but rather to substantially increase the overall proportion of embryos that successfully initiate and sustain early cleavage, a critical prerequisite for successful reprogramming. A previous study examining the effects of TSA on bovine SCNT revealed that the histone deacetylase inhibitor TSA had no impact on the first cleavage stage of SCNT embryos but slightly improved the formation rate of early cleavage-stage embryos (25). Our results are consistent with these findings.

In embryo transfer experiments, owing to the lengthy operational cycles, difficulties in obtaining embryo materials, and developmental delays, reconstructed embryos at the 2-cell stage are typically used for transfer. The ability to develop embryos to the 8-cell stage within a short timeframe for transfer has the potential to enhance embryo implantation rates and nuclear transfer success rates. With rapid advancements in science and technology, gene editing mediated by intracytoplasmic sperm injection (ICSI) has emerged as a superior alternative (26). This technique enables the direct injection of gene editing tools such as CRISPR/Cas9 into the oocyte during sperm injection, simultaneously achieving fertilization and gene editing in a single step. This substantially streamlines the process of generating transgenic organisms and provides new directions and insights for our subsequent research.

Although the small molecule compound significantly enhanced cleavage efficiency in reconstituted embryos, approximately 25% of successfully cleaved embryos exhibited cytoplasmic shrinkage. This phenomenon likely occurs because the drug induced premature entry into the cleavage stage, with subsequent suboptimal culture conditions failing to support normal development.

In recent years, regulating cellular reprogramming capacity and epigenetic modification states using small-molecule compounds has emerged as an effective strategy for enhancing embryonic development efficiency. This study demonstrates that a combination of small molecules, T, EPZ, VPA, Repsox, PD, CHIR, DZNeP, Y, and UNC, can effectively increase the expression of the donor cell histone acetyltransferase Tip60, thereby substantially upregulating pluripotency core genes (NANOG, SOX2, and OCT4) and effectively improving reprogrammed embryo cleavage. This study provides new experimental evidence for exploring small molecules that influence reprogramming and a novel research direction for enhancing embryonic reprogramming efficiency.

## 5 Limitations and perspectives

Chemical reprogramming enhances pluripotency gene expression through histone acetylation; however, the specific targets of action and downstream signaling pathways have not been fully elucidated. Therefore, further studies are needed. This study focused only on the *in vitro* reprogramming efficiency and did not evaluate the *in vivo* developmental potential of SCNT embryos. However, large mammals present disadvantages, such as long cycle times and low fertilization rates, and the survival rate and health status of cloned animals must be subsequently verified through long-term embryo transfer experiments. This may be a key factor in improving the cleavage rate of reconstituted embryo eggs.

## Data Availability

The original contributions presented in the study are included in the article/Supplementary material, further inquiries can be directed to the corresponding authors.

## References

[1] Samiec M Skrzyszowska M. Extranuclear inheritance of mitochondrial genome and epigenetic reprogrammability of chromosomal telomeres in somatic cell cloning of mammals. Int J Mol Sci. (2021) 22:3099. doi: 10.3390/ijms22063099, 33803567 PMC8002851

[2] Jiao D Cheng W Zhang X Zhang Y Guo J Li Z . Improving porcine SCNT efficiency by selecting donor cells size. Cell Cycle. (2021) 20:2264–77. doi: 10.1080/15384101 34583621 PMC8794526

[3] Liu Y Stott R Regouski M Fan Z Perisse IV Patrick T . A retrospective analysis of sheep generated by somatic cell nuclear transfer. Theriogenology. (2024) 227:102–11. doi: 10.1016/j.theriogenology 39047406

[4] Malin K Witkowska-Piłaszewicz O Papis K. The many problems of somatic cell nuclear transfer in reproductive cloning of mammals. Theriogenology. (2022) 189:246–54. doi: 10.1016/j.theriogenology.2022.06.030, 35809358

[5] Luo T. A small-molecule approach towards the fountain of youth: chemically induced pluripotent stem cells. Natl Sci Rev. (2022) 9:nwac181. doi: 10.1093/nsr/nwac181, 36452429 PMC9701096

[6] Chen R Xie W Cai B Qin Y Wu C Zhou W . Establishment and identification of a CiPSC lineage reprogrammed from FSP-tdTomato mouse embryonic fibroblasts (MEFs). Stem Cells Int. (2018) 2018:5965727. doi: 10.1155/2018/5965727, 30675169 PMC6323470

[7] van der Zanden SY Luimstra JJ Neefjes J Borst J Ovaa H. Opportunities for small molecules in cancer immunotherapy. Trends Immunol. (2020) 41:493–511. doi: 10.1016/j.it.2020.04.004, 32381382

[8] Zhe X Ma H Zhang W Ding R Hao F Gao Y . Scriptaid improves cashmere goat embryo reprogramming by affecting donor cell pluripotency molecule NANOG expression. Animals. (2025) 15:1022. doi: 10.3390/ani15071022, 40218415 PMC11988105

[9] Hou P Li Y Zhang X Liu C Guan J Li H . Pluripotent stem cells induced from mouse somatic cells by small-molecule compounds. Science. (2013) 341:651–4. doi: 10.1126/science.1239278, 23868920

[10] Selokar NL St John L Revay T King WA Singla SK Madan P. Effect of histone deacetylase inhibitor valproic acid treatment on donor cell growth characteristics, cell cycle arrest, apoptosis, and handmade cloned bovine embryo production efficiency. Cell Reprogram. (2013) 15:531–42. doi: 10.1089/cell.2013.0018, 24180742

[11] Matoba S Shikata D Shirai F Tatebe T Hirose M Nakata A . Reduction of H3K9 methylation by G9a inhibitors improves the development of mouse SCNT embryos. Stem Cell Reports. (2024) 19:906–21. doi: 10.1016/j.stemcr.2024.04.003, 38729154 PMC11390627

[12] Guan J Wang G Wang J Zhang Z Fu Y Cheng L . Chemical reprogramming of human somatic cells to pluripotent stem cells. Nature. (2022) 605:325–31. doi: 10.1038/s41586-022-04593-5, 35418683

[13] Li H Guan W Huang J Shen P Wu J Luo H . A complete model of mouse embryogenesis through organogenesis enabled by chemically induced embryo founder cells. Cell. (2025) 188:5912–5930.e20. doi: 10.1016/j.cell.2025.07.018, 40780195

[14] Ladewig J Mertens J Kesavan J Doerr J Poppe D Glaue F . Small molecules enable highly efficient neuronal conversion of human fibroblasts. Nat Methods. (2012) 9:575–8. doi: 10.1038/nmeth.1972, 22484851

[15] Boland MJ Nazor KL Loring JF. Epigenetic regulation of pluripotency and differentiation. Circ Res. (2014) 115:311–24. doi: 10.1161/CIRCRESAHA.115.301517, 24989490 PMC4229506

[16] Yu J Vodyanik MA Smuga-Otto K Antosiewicz-Bourget J Frane JL Tian S . Induced pluripotent stem cell lines derived from human somatic cells. Science. (2007) 318:1917–20. doi: 10.1126/science.1151526, 18029452

[17] Wu K Fan D Zhao H Liu Z Hou Z Tao W . Dynamics of histone acetylation during human early embryogenesis. Cell Discov. (2023) 9:29. doi: 10.1038/s41421-022-00514-y, 36914622 PMC10011383

[18] Jafarpour F Ghazvini Zadegan F Ostadhosseini S Hajian M Kiani-Esfahani A Nasr-Esfahani MH. siRNA inhibition and not chemical inhibition of Suv39h1/2 enhances pre-implantation embryonic development of bovine somatic cell nuclear transfer embryos. PLoS One. (2020) 15:e0233880. doi: 10.1371/journal.pone.0233880, 32497112 PMC7272017

[19] Zhao Y Zhao T Guan J Zhang X Fu Y Ye J . A xen-like state bridges somatic cells to pluripotency during chemical reprogramming. Cell. (2015) 163:1678–91. doi: 10.1016/j.cell.2015.11.017, 26686652

[20] Wang J Sun S Deng H. Chemical reprogramming for cell fate manipulation: methods, applications, and perspectives. Cell Stem Cell. (2023) 30:1130–47. doi: 10.1016/j.stem.2023.08.001, 37625410

[21] Li W Ding S. Small molecules that modulate embryonic stem cell fate and somatic cell reprogramming. Trends Pharmacol Sci. (2010) 31:36–45. doi: 10.1016/j.tips.2009.10.002, 19896224

[22] Matoba S Zhang Y. Somatic cell nuclear transfer reprogramming: mechanisms and applications. Cell Stem Cell. (2018) 23:471–85. doi: 10.1016/j.stem.2018.06.018, 30033121 PMC6173619

[23] Boyer LA Lee TI Cole MF Johnstone SE Levine SS Zucker JP . Core transcriptional regulatory circuitry in human embryonic stem cells. Cell. (2005) 122:947–56. doi: 10.1016/j.cell.2005.08.020, 16153702 PMC3006442

[24] Cheng L Wang Y Guan J Deng H. Decoding human chemical reprogramming: mechanisms and principles. Trends Biochem Sci. (2025) 50:520–31. doi: 10.1016/j.tibs.2025.03.004, 40169299

[25] Akagi S Matsukawa K. Effects of trichostatin a on the timing of the first cleavage and in vitro developmental potential of bovine somatic cell nuclear transfer embryos. Cell Reprogram. (2022) 24:142–9. doi: 10.1089/cell.2022.0003, 35404091

[26] Jamal MA Husnain A Xu K Wei HJ. Factors affecting the intracytoplasmic sperm cell injection outcomes: a meta-analysis of porcine studies. J Adv Res. (2025) 78:757–76. doi: 10.1016/j.jare.2025.02.040, 40032025 PMC12684963

