# Supplementary material for: Chemical reprogramming regulates Tip60 expression to improve cleavage rates in somatic cell nuclear transfer reconstituted embryos of cashmere goats
Source: Front Vet Sci. 2025 Dec 19;12:1720533. doi: 10.3389/fvets.2025.1720533 (PMC12758406; doi:10.3389/fvets.2025.1720533)
Supplement: Supplementary file 1 [file Table_1.docx]

**Supplementary Information**

**Table S1 Primer sequences**

| Gene Name | Sequence | Product Length (bp) |
| --- | --- | --- |
| *GAPDH* | F 5' GGTCGGAGTGAACGGAT 3'  R 5' TCTGCCTTGACTGTGCC 3' | 100 |
| *OCT 4* | F 5' CCAGAAGGGCAAACGAT 3'  R 5' GGAATGGGACCGAAGAGTA 3' | 98 |
| *HAT1* | F 5' TTACAGCGGAAGATCCAT 3'  R 5' TCATAAACCCGTCTAGCAT 3' | 90 |
| *CBP* | F 5’ CTTGGACTCCCCTACCTG 3’  R 5’ TGATGGCTGCTGATCTGT 3’ | 166 |
| *Tip60* | F 5’ CGAAGCTACTGGTCCCAGAC 3’  R 5’ CCTCCGACAGGGTGAGGATA 3’ | 193 |
| *PCAF* | F 5’ CAGGGGTGTGATCGAGTTCC 3’  R 5’ GGGTTTTGTGTTTCGGGTCG 3’ | 167 |
| *P300* | F 5' TGAGAAGCAGCCTTCACAGG 3’  R 5’ TCGCTGAAGTACTTGGCTGG 3’ | 200 |
| *NANOG* | F 5' GCAACGGCAGAATACCC 3'  R 5' CATTGATTGTTCCAAGGCT 3' | 100 |
| *SOX 2* | F 5' CATGCACCGCTACGACG 3'  R 5' GCCCTGCTGAGAATAGGACA 3' | 88 |
| *Ki67* | F 5' CCCAGTATTAAAATGAGCG 3'  R 5' TGATGTTGCCTTTGGAGTC 3' | 92 |
| *PCNA* | F 5' CTTGAAGAAAGTGCTGGAG 3'  R 5' TGGACATGCTGGTGAGG 3' | 93 |

**Table S2 Key resources table**

| **Reagent or resource** | **Source** | **ID** |
| --- | --- | --- |
| Cell lineage |  |  |
| Goat fetal fibroblasts cells | Arbas Cashmere goat (45–90 d) | N/A |
| Antibodies |  |  |
| Alpha Tubulin Polyclonal antibody | Proteintech, Wuhan, China | 16007-1-AP |
| [Tip60 Rabbit pAb](https://www.immunoway.com.cn/products/primary-antibodies/YT6133-Tip60-Rabbit-pAb.html) | Immunoway, JiangSu, China | YT6133 |
| HRP-conjugated AffiniPure Goat Anti-Rabbit (H+L) | Proteintech, Wuhan, China | SA00001-2 |
| Oligonucleotides |  |  |
| PrimeScript™ RT reagent Kit with gDNA Eraser (Perfect Real Time) | Takara, Dalian, China | RR047A |
| TB Green® Premix Ex Taq™ II (Tli RNaseH Plus) | Takara, Dalian, China | RR820A |
| Cellular molecular reagents |  |  |
| DMEM/F12 1:1 mixture | Vivacell, Shanghai, China | C3130-0500 |
| Dulbecco's Phosphate Buffered Saline (DPBS) | Vivacell, Shanghai, China | C3590-0500 |
| Certified Fetal Bovine Serum | Vivacell, Shanghai, China | C04001-500 |
| Trypsin EDTA Solution A (0.25%) | Vivacell, Shanghai, China | C3530-0500 |
| Tranyloypromine (T) | MCE, Shanghai, China | HY-B1496 |
| EPZ004777 (EPZ) | Yeasen, Shanghai, China | 53598ES08 |
| Valproic acid (VPA) | Yeasen, Shanghai, China | 52983ES70 |
| 616425 (Repsox) | Yeasen, Shanghai, China | 53016ES08 |
| PD0325901 (PD) | Yeasen, Shanghai, China | 53011ES08 |
| CHIR99021 (CHIR) | Yeasen, Shanghai, China | 53003ES10 |
| 3-deaEaneplanocin A (DZNeP) | Yeasen, Shanghai, China | 53282ES03 |
| Y-27632(Y) | Yeasen, Shanghai, China | 52983ES70 |
| UNC0379(UNC) | Yeasen, Shanghai, China | 53597ES08 |
| CCK8- Assay Kit | Yeasen, Shanghai, China | 40203ES60 |
| RNAiso Plus | Takara, Dalian, China | 9109 |
| DMSO | Sigma-Aldrich, USA | D2650 |
| Bovine Serum Albumin | Sigma-Aldrich, USA | [A1933](https://www.sigmaaldrich.cn/CN/zh/product/sigma/a1933) |
| ECL Western Blotting Substrate | Thermo Fisher Scientific, Waltham, MA, USA | 32109 |
| Pierce® BCA Protein Assay Kit | Thermo Fisher Scientific, Waltham, MA, USA | 23227 |
| PageRuler Prestained Protein Ladder | Thermo Fisher Scientific, Waltham, MA, USA | 26616 |
| Mammalian Protein Extraction Kit | CWBIO, Beijing, China | CW0889M |
| Medium 199 (1X), liquid | Gibco, NY, USA | 11150059 |
| Hyaluronidase | Sigma-Aldrich, USA | H1115000 |
| Cytochalasin B | Sigma-Aldrich, USA | C2743 |
| A23187 | Sigma-Aldrich, USA | C7522 |
| Software and algorithms |  |  |
| Adobe Illustrator 2025 | Adobe | https://www.adobe.com/ |
| GraphPad Prism v10 | GraphPad Software | https://www.graphpad.com/ |
| ImageJ v1.8 | Schneider et al., 2012 | https://imagej.nih.gov/ij |
